# The Enteropathogenic *E. coli* (EPEC) Tir Effector Inhibits NF-κB Activity by Targeting TNFα Receptor-Associated Factors

**DOI:** 10.1371/journal.ppat.1002414

**Published:** 2011-12-01

**Authors:** Marie-Hélène Ruchaud-Sparagano, Sabrina Mühlen, Paul Dean, Brendan Kenny

**Affiliations:** Institute for Cell and Molecular Biosciences, Medical School, Newcastle University, Newcastle-upon-Tyne, United Kingdom; Stanford University School of Medicine, United States of America

## Abstract

Enteropathogenic *Escherichia coli* (EPEC) disease depends on the transfer of effector proteins into epithelia lining the human small intestine. EPEC E2348/69 has at least 20 effector genes of which six are located with the effector-delivery system genes on the Locus of Enterocyte Effacement (LEE) Pathogenicity Island. Our previous work implied that non-LEE-encoded (Nle) effectors possess functions that inhibit epithelial anti-microbial and inflammation-inducing responses by blocking NF-κB transcription factor activity. Indeed, screens by us and others have identified novel inhibitory mechanisms for NleC and NleH, with key co-operative functions for NleB1 and NleE1. Here, we demonstrate that the LEE-encoded Translocated-intimin receptor (Tir) effector has a potent and specific ability to inhibit NF-κB activation. Indeed, biochemical, imaging and immunoprecipitation studies reveal a novel inhibitory mechanism whereby Tir interaction with cytoplasm-located TNFα receptor-associated factor (TRAF) adaptor proteins induces their proteasomal-independent degradation. Infection studies support this Tir-TRAF relationship but reveal that Tir, like NleC and NleH, has a non-essential contribution in EPEC's NF-κB inhibitory capacity linked to Tir's activity being suppressed by undefined EPEC factors. Infections in a disease-relevant intestinal model confirm key NF-κB inhibitory roles for the NleB1/NleE1 effectors, with other studies providing insights on host targets. The work not only reveals a second Intimin-independent property for Tir and a novel EPEC effector-mediated NF-κB inhibitory mechanism but also lends itself to speculations on the evolution of EPEC's capacity to inhibit NF-κB function.

## Author Summary

Enteropathogenic *Escherichia coli* (EPEC) remain an important cause of infant diarrhoea and death in developing countries. Undoubtedly, a key pathogenic event relates to the bacteria's ability to inhibit the expression of anti-microbial and inflammation-inducing molecules regulated by the NF-κB transcription factor. This inhibitory process was previously linked to undefined EPEC effectors blocking NF-κB function. In this manuscript, we report that the most extensively studied EPEC effector, Tir, possesses a potent and specific ability to inhibit the cytokine, TNFα from activating NF-κB. Moreover, this finding leads to the discovery of a novel inhibitory mechanism relating to the induced degradation of the TNFα Receptor-Associated Factor (TRAF) adaptor protein. Additional work supports reported NF-κB inhibitory activities for multiple non-LEE-encoded effectors and key roles for two (identifying possible target proteins) leading to speculations on the evolution of EPEC's multi-effector strategy for inhibiting NF-κB activity.

## Introduction

The EPEC disease process depends on a protein delivery system, encoded by the Locus of Enterocyte Effacement (LEE) Pathogenicity Island, that transfers effector proteins directly into the cytoplasm of infected epithelia [1]–[3]. This delivery apparatus is composed of a Type Three Secretion System (T3SS) and a filamentous extension - formed by the EPEC secreted/signalling protein A (EspA) tipped by EspB/EspD to form a pore in the host plasma membrane - generating a conduit for transferring effectors into host cells [1], [3]. The LEE region also encodes other factors, including the bacterial Intimin surface protein and six effectors: Translocated-Intimin receptor (Tir), Mitochondrial-associated protein (Map), EspF, EspG, EspH and EspZ (with EspB also exhibiting effector functions) [1], [3]. Prototypic EPEC (E2348/69) has at least fourteen additional non-LEE-encoded (Nle or Esp nomenclature) effector genes distributed on six horizontally-acquired mobile genetic elements [3], [4].

The EPEC disease process is characterised by a number of histo-pathological events including (i) initial non-intimate attachment to epithelial cells, (ii) bacteria sinking into the microvillus surface, (iii) intimate interaction with the host plasma membrane, (iv) nucleation of actin beneath intimately-adherent bacteria and (v) extensive loss/effacement of microvilli [1]–[3]. Studies with the Caco2 small intestinal model have provided insights on these events and revealed a plausible mechanism to explain the rapid onset of EPEC-induced watery diarrhoea [5]. Moreover, the use of such models have uncovered EPEC's ability to disrupt cell-cell interactions [5]–[7] subsequently verified by *in vivo* studies [8], [9]. While disruption of cell-cell interactions is an inflammatory event, human EPEC infections are normally associated with unexpectedly weak inflammation [2] thereby suggesting that the pathogen employs inhibitory mechanisms. Indeed, studies have revealed that EPEC inhibits Nuclear Factor κB (NF-κB) function - responsible for inducing the expression of anti-microbial and inflammation-related molecules - before barrier function is disrupted [10].

**Figure 1:**
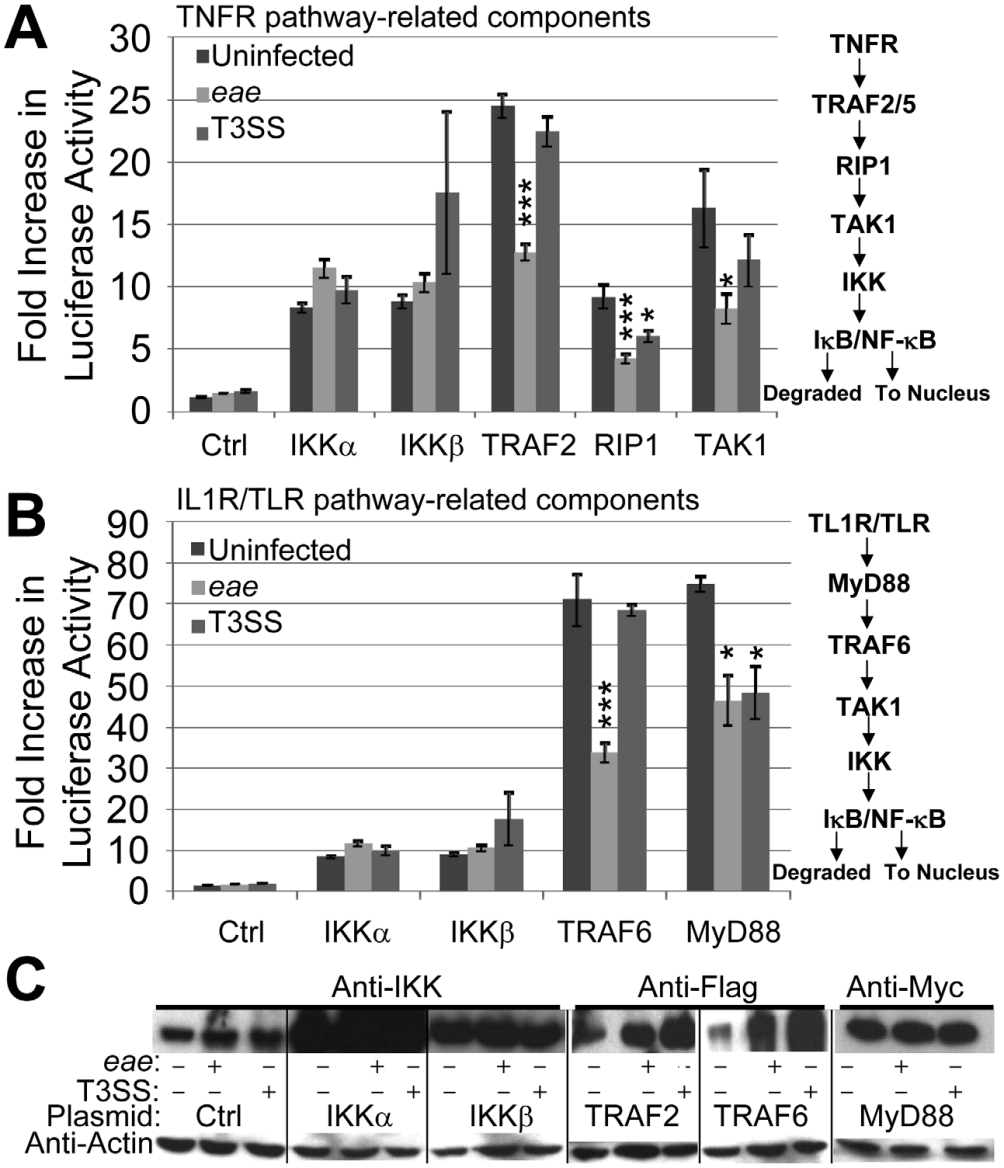
EPEC T3SS-dependent inhibition of NF-κB activity is associated with effectors targeting pathway components upstream of the IKK complex. HeLa cells were co-transfected with vectors encoding luciferase under the transcriptional control of NF-κB and the indicated components of the TNFα (A) and IL1R/TLR (B) signalling pathways. Ctrl refers to control empty vector with IKK data in A and B from the same experiment illustrating scale differences. Cells were left uninfected or incubated for 3 hours with *eae* (Intimin-deficient) or effector delivery-defective (T3SS) mutants prior to (A & B) assaying cellular luciferase activity or (C) processing for Western blot analysis probing for cellular levels of actin (loading control) and indicated signalling components. Fold increase in luciferase activity data is mean (+/- S.D.) of three independent experiments (done in duplicate) with significance (one-way ANOVA; *p≤0.05, ***p≤0.005) shown relative to corresponding uninfected control.

Host cell detection of foreign antigens (by Toll-like receptors; TLR) and cytokines (such as TNFα and IL1β by TNFR and IL1R, respectively) triggers a cascade of phosphorylation and ubiquitination events leading to IKK (Inhibitor of KappaB kinase) complex activation [11]–[13]. The activated IKK complex, composed of two kinases (IKKα, IKKβ and a regulatory subunit (NEMO/IKKγ), phosphorylates IκB (Inhibitor of κB) to induce its proteasomal-dependent degradation thereby releasing NF-κB for import into the nucleus to transcribe genes [14]. NF-κB function is regulated through many mechanisms including IκB re-synthesis, modification of NF-κB (or accessory factors) and altering NF-κB access to promoters [13]–[15]. In addition, NF-κB activity can be regulated at the level and/or function of signalling pathway components that includes kinases, phosphatases, ubiquitin ligases, de-ubiquitinases and adaptor proteins [13], [16]–[18]. TLR, IL1R and TNFR signalling to the IKK complex depends on TNFα Receptor-Associated Factor (TRAF) adaptor proteins and TGF**β**-Activating Kinase 1 (TAK1) with pathway-specific components including kinases such as Receptor-Interacting Protein1 (RIP1) and adaptors such as Myeloid Differentiation primary response gene (88) (MyD88) [13], [18]–[20](see Fig. 1).

EPEC inhibition of NF-κB activity triggered by flagellin (recognised by TLR5) or cytokines (including TNFα and IL1β) in the Caco-2 small intestinal model depends on the pathogen possessing a functional effector-delivery system [10], [21]. Previous work has argued against a need for all LEE effectors, Intimin and a subset of Nle effectors (Orf3/EspG2, NleA, NleF and NleH) in this EPEC NF-κB inhibitory process thereby implicating other Nle effectors [10]. Indeed, screening programs by us and others have revealed novel NF-κB inhibitory activities for NleC and NleH with critical co-operative roles for NleE1 and NleB1 [22]–[29]. Here, we show that the LEE-encoded Translocated-Intimin receptor has a potent and specific Intimin-independent ability to inhibit NF-κB activation leading to the discovery of a novel inhibitory mechanism. Moreover, our work provides insights on the NleE1/NleB1 subversive process and on the possible evolution of EPEC's capacity to inhibit NF-κB activity.

## Results

### EPEC inhibits NF-κB activation by targeting components at or upstream of IKKα and IKKβ

EPEC requires a functional T3SS to inhibit antigens (such as flagellin) and cytokines (such as TNFα and IL1β) from activating NF-κB [10], [21]. To gain insight on the inhibitory mechanism, we examined EPEC's ability to interfere with NF-κB activity driven by plasmid-expression of TLR/IL1R and TNFR signalling pathway components (see Fig. 1). Thus, HeLa cells were co-transfected with plasmids expressing luciferase under the transcriptional control of NF-κB - via five repeats of the κB consensus promoter [30] - and specific signalling pathway components [30]–[32] prior to infecting with EPEC strains and quantifying luciferase cellular activity (see Materials and Methods). As EPEC can induce HeLa cells detachment [33], we employed the *eae* mutant (which lacks the Intimin surface protein) that, like wild type EPEC, inhibits NF-κB activity [10] but has little capacity to detach HeLa cells (Quitard *et al*., unpublished). The luciferase assay revealed basal NF-κB activity within uninfected HeLa cells as reported [23], with no significant change following infection with the *eae* or T3SS (effector-delivery defective) strains (Fig. 1A). Plasmid expression of TNFR pathway-related kinase (IKKα, IKKβ, TAK1 and RIP1) or adaptor (TRAF2) proteins increased cellular luciferase levels by 8 to 24 fold. Interestingly, the T3SS mutant inhibited increases driven by plasmid-expressed RIP-1 (Fig. 1A) supporting the presence of T3SS-independent inhibitory mechanisms in the HeLa model [28], [29]. By contrast, the *eae* mutant infection inhibited luciferase activity driven by plasmid-expression of all components except IKKα and IKKβ (Fig. 1A). Parallel studies on TLR/IL1R pathway components revealed another T3SS-independent inhibitory mechanism relating to MyD88 (Fig. 1B), with the *eae* mutant inhibiting signalling driven by plasmid-expressed TRAF6 and MyD88 (Fig. 1B). Western blot analyses verified the T3SS-dependent delivery of effectors (EspF and Tir; not shown) and plasmid-expression of examined host proteins, with the latter revealing unexpected infection-related increases in the cellular level of Flag-tagged TRAF proteins (Fig. 1C). Thus, consistent with previous studies [28], [29], EPEC infection of HeLa cells inhibits signalling to NF-κB by T3SS-independent and -dependent mechanisms. Moreover, the work implies that T3SS-dependent inhibitory mechanism(s) relates to effector(s) acting at or upstream of the IKKα/β complex.

### The Translocated Intimin receptor inhibits TNFα-induced NF-κB activation

**Figure 2:**
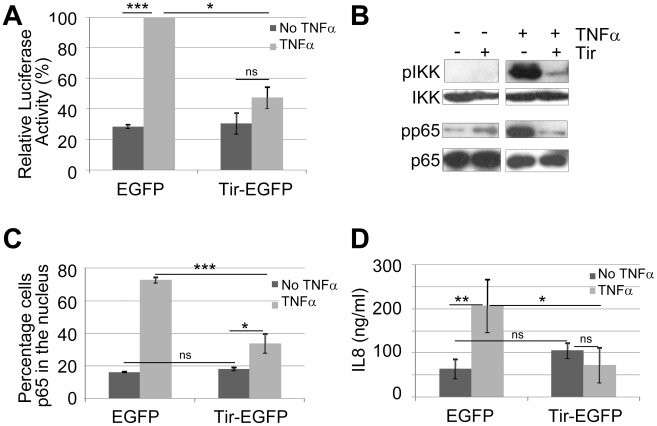
Ectopically-expressed Tir is a potent inhibitor of TNFα-induced NF-κB activity. HeLa cells transfected with vectors encoding eGFP or Tir-eGFP were treated (light grey shading or +) or not (dark grey shading or -) with TNFα prior to processing for (A) luciferase activity - these experiments involved co-transfection with the NF-κB reporter vector, (B) Western Blot analysis probing for total and phosphorylation-associated activated form of IKKα/β and p65 component of NF-κB, (C) microscopy-based quantification of the percentage of cells with p65 within the nucleus (counting a minimum of 50 cells in three independent experiments), and (D) secreted levels of IL8 - a NF-κB dependent gene product. Graphical data is mean (+/- S.D.) of three independent experiments (in duplicate for luciferase and IL8 assays) with significance (one-way ANOVA; *p≤0.05, **p≤0.01, ***p≤0.005, ns - not significant) shown between indicated data sets.

To identify Nle effectors postulated to inhibit NF-κB activity [10], putative *nle* effector genes [3], [4] were cloned into mammalian expression vectors and co-transfected into HeLa cells with the NF-κB luciferase reporter vector for screening. Indeed, this approach identified a NF-κB inhibitory activity for NleC leading to its definition as a zinc metalloprotease that degrades NF-κB complexes [23] as supported by independent studies [22], [24], [25]. Interestingly, inclusion of an available Tir-expressing construct [34] in the screening program indicated that this LEE effector could prevent TNFα from activating NF-κB. To investigate this putative NF-κB inhibitory activity in more detail, the *tir* gene was sub-cloned into *pEGFP*-N1 to generate a Tir-eGFP fusion protein with *pEGFP*-N1 serving as a negative control. Fig. 2A reveals similar basal luciferase activity in cells transfected with p*EGFP* or p*tir*-*EGFP* plasmids, with TNFα leading to a significant increase in NF-κB reporter activity for p*EGFP*, but not p*tir*-*EGFP* transfected cells. The relevance of this finding to NF-κB function was illustrated by Western blot analysis where expression of Tir-eGFP, unlike eGFP, inhibited TNFα from inducing the phosphorylation-associated activation of IKKα/β kinases and the NF-κB component, p65 (Fig. 2B). Inhibition specificity was illustrated by unaltered total cellular levels of IKKα/β and p65 proteins (Fig. 2B). Furthermore, fluorescent microscopy examinations revealed p65 within the nucleus of ∼18% of eGFP or Tir-eGFP expressing cells, with TNFα treatment increasing this to ∼70% for eGFP expressing cells but only ∼35% for Tir-eGFP expressing cells (Fig. 2C). As IL8 secretion requires NF-κB activity [35], we examined the extra-cellular levels of this chemokine (see Materials and Methods). Consistent with the luciferase NF-κB reporter data (Fig. 2A), *pEGFP* and *ptir-EGFP* transfected cells released similar basal levels of IL8, with TNFα treatment increasing IL8 secretion levels from *pEGFP* but not p*tir*-*EGFP* transfected cells (Fig. 2D). Thus, expressing Tir-eGFP within HeLa cells specifically prevents TNFα from transducing signals that activate NF-κB in a manner linked to a blockage in the phosphorylation-associated activation of IKK components needed to release NF-κB for nuclear import. The absence of other EPEC factors in these experiments illustrate that this novel property of the Translocated-Intimin receptor (Tir) effectors occurs independently of Intimin.

### Tir provides Yersinia with an EPEC-like capacity to inhibit TNFα-induced IL8 secretion

**Figure 3:**
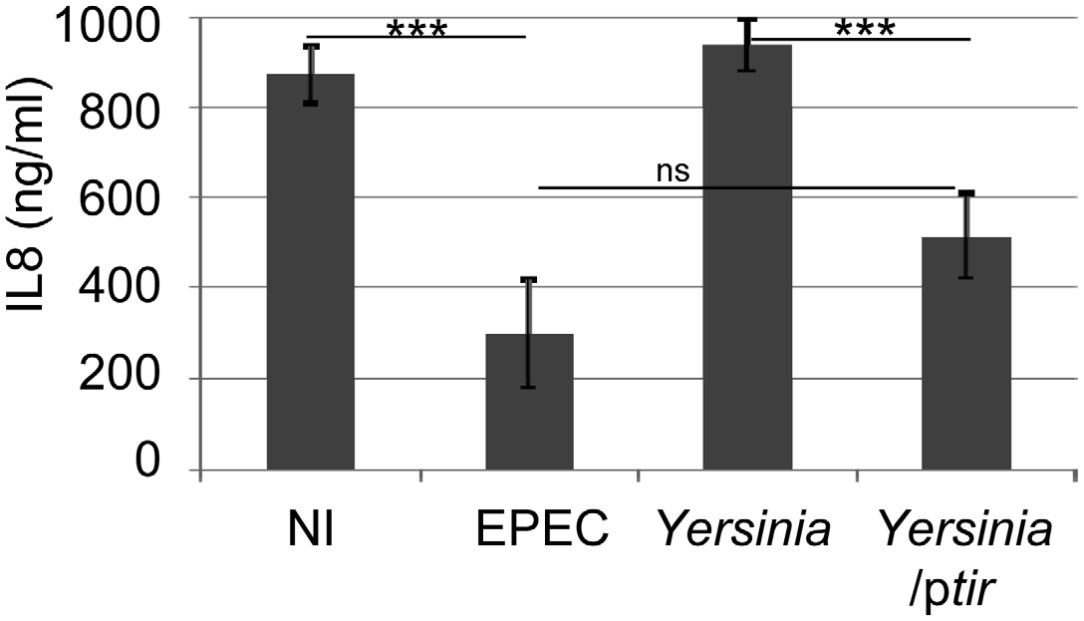
*Yersinia*-delivered Tir inhibits TNFα-induced IL8 secretion to a similar extent as EPEC-delivered effectors. HeLa cells were left uninfected (NI) or incubated with EPEC or a *Yersinia pseudotuberculosis* (Yop effector-deficient) strain carrying or not a Tir-encoding plasmid (p*tir*) prior to killing the bacteria, adding TNFα and quantifying IL8 secretion levels. IL8 data is mean (+/- S.D.) of three independent experiments (done in duplicate), with significance (one-way ANOVA; ***p≤0.005, ns - not significant) shown between indicated data sets.

To support the specific and Intimin-independent nature of the Tir inhibitory activity, studies evaluated Tir's ability to block TNFα-induced IL8 secretion following its delivery into HeLa cells by *Yersinia pseudotuberculosis* as previously described [34]. Importantly, the control Tir-negative *Yersinia* strain (which lacks most of its own T3SS-delivered effectors) failed to inhibit TNFα-induced IL8 secretion, whereas the Tir-expressing variant inhibited this process to a similar degree as EPEC-delivered effectors (Fig. 3). Western blot analyses verified *Yersinia*-delivery of Tir where it underwent partial host kinase-mediated modification, compared to EPEC-delivered Tir (not shown), as previously described [34]. Given that TNFα augmentation of IL8 secretion requires NF-κB activity [35], this work supports the premise that Tir (in the absence of other EPEC factors, including Intimin) possesses a potent and specific ability to prevent TNFR-induced signalling from activating NF-κB.

### Tir inhibition of NF-κB activity is associated with its targeting of the TRAF2 adaptor protein

**Figure 4:**
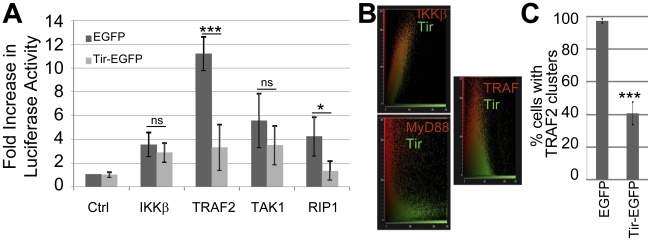
Tir inhibition of NF-κB activation is associated with Tir targeting TRAF proteins. (A) HeLa cells were co-transfected with vectors encoding luciferase under the transcriptional control of NF-κB and indicated components of the TNFR pathway prior to quantifying cellular luciferase levels. Ctrl refers to control empty vector. Fold increase in luciferase activity data is mean (+/- S.D.) of three independent experiments (done in duplicate) with significance (one-way ANOVA; *p≤0.05, ***p≤0.005, ns - not significant) shown relative to corresponding control. In (B) HeLa cells were co-transfected with vectors encoding Tir-eGFP with either epitope-tagged IKKβ, MyD88 or TRAF2 proteins prior to fixing cells (∼24 hour post-transfection) and probing the cellular location of the plasmid-expressed proteins (using appropriate primary and different fluorescent-conjugated secondary antibodies). The host protein (Red) and Tir-eGFP (Green) signals collected from multiple serial slices through a field of view (∼20 cells) were obtained and plotted to display the degree of overlap. (C) Quantification of the percentage of HeLa cells containing Flag-tagged TRAF2 clusters in eGFP and Tir-eGFP expressing cells. Data is mean (+/- S.D.) of three independent experiments examining ∼50 cells per experiment (one-way ANOVA; ***p≤0.005).

To gain insight on how Tir inhibits TNFα-induced NF-κB activation, HeLa cells were co-transfected with plasmids encoding (i) the NF-κB luciferase reporter protein, (ii) TNFR signalling pathway components and (iii) eGFP or Tir-eGFP proteins prior to assaying cellular luciferase levels. This work revealed that Tir-eGFP, but not eGFP, inhibited luciferase activity driven by plasmid-expression of TRAF2 and RIP1, but not TAK1, IKKβ (Fig. 4A) or IKKα (not shown). Fluorescence microscopy studies were undertaken to determine the cellular location of over-expressed signalling components and to assess if Tir-eGFP expression induced detectable changes. Staining for plasmid-expressed Tir and IKK kinase proteins revealed diffuse cytoplasmic signals in contrast to cytoplasmic aggregates/clusters for the TRAF2 (Fig. 4B and Fig. S1) and MyD88 (not shown) adaptor proteins. Cytoplasmic clustering of plasmid-expressed TRAF2 has been reported [36]. Imaging of co-transfected cells revealed similar, distinct and partially-overlapping signals for Tir/IKK, Tir/MyD88 and Tir/TRAF signals, respectively (Fig. 4B and Fig. S1). Intriguingly, Tir-eGFP expression was associated with a loss of TRAF clusters (Fig. S1B) as supported by quantification studies (Fig. 4C). Thus, Tir may inhibit plasmid-expressed TRAF2 from transmitting signalling to NF-κB by inducing the disaggregation and/or degradation of activation-associated clusters.

### Tir interaction with TRAF2 is associated with the proteasomal-independent degradation of the adaptor protein

**Figure 5:**
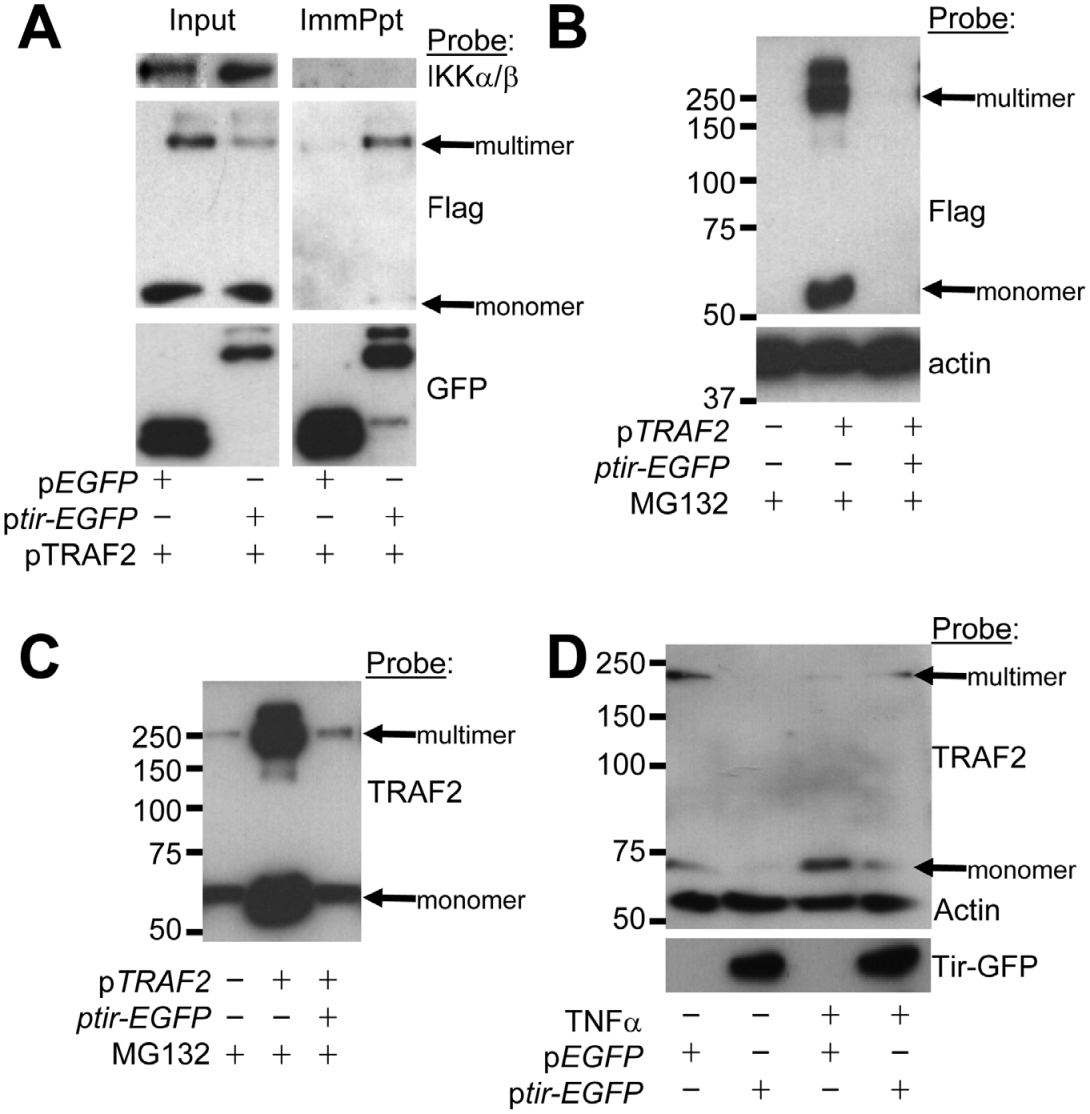
Tir interacts with TRAF2 to induce the latter's proteasomal-independent degradation. HeLa cells were co-transfected with vectors encoding (i) Flag-tagged TRAF2 and (ii) eGFP or Tir-eGFP proteins prior to (A) isolating Triton-X100 soluble host fractions - taking an ‘input’ reference sample - with the remaining pool used to isolate GFP proteins (ImmPpt) using GFP-Trap beads. In (B) and (C) HeLa cells were pre-treated with the proteasome inhibitor, MG132, before and following transfection of Tir-eGFP and Flag-tagged TRAF2 plasmids, with total cellular samples isolated 24 hr post-transfection. In (D) HeLa cells were left untransfected or transfected with p*EGFP* or p*tir-EGFP* plasmids and (24 hrs post-transfection) incubated in the presence or absence of TNFα (30 min) prior to lysis. Samples were subjected to Western blot analysis probing with anti-IKK, FLAG, GFP, TRAF2 and/or actin antibodies.

To examine predicted Tir-TRAF interactions, GFP-Trap beads were used to isolate eGFP and Tir-eGFP proteins from cells co-transfected with the Flag-tagged TRAF2 expressing plasmid. Fig. 5A reveals eGFP and Tir-eGFP within input cellular extracts and their isolation by the GFP-Trap beads. While the IKKα/β proteins were present in the input pool, they did not co-isolate with eGFP or Tir-eGFP (Fig. 5A). Probing for Flag-tagged TRAF2 revealed a prominent monomer-sized band with smaller amounts of a trimer-sized TRAF species in the input pool (Fig. 5A). TRAF2 function is linked to the formation of homo- or hetero-trimers [13], [37]. Intriguingly, the minor trimer-sized TRAF2 species preferentially isolated with Tir-eGFP, though some monomer was co-isolated (Fig. 5A). This work suggests that Tir interacts (either directly or indirectly) with the activation-associated multimeric form of TRAF adaptor proteins.

Examination of the input samples (Fig. 5A) suggested that Tir expression may decrease the cellular levels of multimeric (and perhaps monomeric) Flag-tagged TRAF2 protein (Fig. 5A). This premise was supported by demonstrating that co-expression of Tir-eGFP with Flag-tagged TRAF2 could lead to the complete loss of TRAF2 from cell extracts, while the actin loading control protein remained unchanged (Fig. 5B). As inflammatory signalling is commonly regulated by targeting components for proteasomal-dependent degradation [38], we used the proteasomal inhibitor MG132. Whilst MG132 inhibitory activity was confirmed, as per a parallel study [23], it failed to prevent the Tir-mediated loss of Flag-tagged TRAF2 proteins (Fig. 5B). Interestingly, probing the fate of endogenous TRAF2 suggested that it is not a substrate for Tir degradation, at least in cells expressing the Flag-tagged TRAF2 variant. Thus, similar levels of monomer and trimer-sized TRAF2 bands were evident in non-transfected and p*tir-EGFP* transfected cells (Fig. 5C) while the more prominent bands in p*TRAF2*-transfected cells correspond to the Flag-tagged variant (Fig. 5C versus 5B).

To investigate whether endogenous TRAF2 is a substrate for Tir-induced degradation, its fate was examined in cells that express Tir-eGFP, but not Flag-tagged TRAF2 proteins. Fig. 5D reveals that Tir expression can, in fact, induce the cellular loss of endogenous TRAF2. Interestingly, TNFα treatment of control cells reduced the level of multimeric TRAF2 with an increase in the monomer species (Fig. 5D) that, presumably, reflects intrinsic host mechanism(s) for down-regulating cytokine-induced signalling. By contrast, TNFα treatment of p*tir-EGFP* transfected cells produced a small pool of TRAF2 protein (monomer and trimer-sized forms; Fig. 5D) that may explain why TNFα triggered some p65 relocation to the nucleus of Tir-eGFP expressing cells (Fig. 2C). Ubiquitin-modified TRAF2 plays a key role in activating RIP1 which activates TAK1 [39] to, perhaps, explain why Tir inhibits NF-κB luciferase activity driven by plasmid-expressed RIP1 and, to a lesser extent, TAK1 (Fig. 4A). Collectively, the work implies that Tir inhibits TNFα-induced NF-κB activation by interacting (directly or indirectly) with TRAF2 - a key component of the TNFR signalling pathway - to induce its proteasomal-independent degradation.

### Tir has a non-essential contributory role in enabling EPEC to inhibit TNFα-induced NF-κB activity

**Figure 6:**
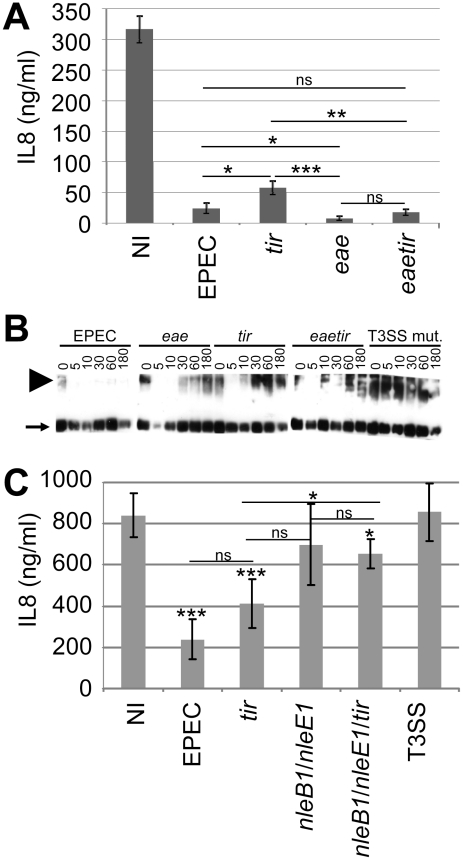
Non-essential contribution of Tir in the EPEC NF-κB inhibitory process. In (A) HeLa cells were infected for 3 hrs with indicated strains prior to killing the bacteria, adding TNFα (30 minutes) and quantifying of IL8 secretion levels. Data is mean (+/- S.D.) of three independent experiments (done in duplicates) with significance (one-way ANOVA; *p≤0.05, **p≤0.01, ***p≤0.005, ns - not significant) shown between indicated pairs. In (B) HeLa cells were left uninfected or infected with indicated strains (using gentle centrifugation - 500 xg, 5 minutes - to synchronise infections) and cellular samples isolated at indicated time-points for Western blot analysis probing with anti-TRAF2 antibodies. Arrow and arrowhead, respectively, indicate monomeric and activation-associated trimeric TRAF2 forms. In (C) polarised Caco2 cells were apically-infected for 3 hours with indicated strains prior to killing the bacteria, adding TNFα (30 minutes) to the baso-lateral surface and assaying IL8 secretion levels. Data is mean (+/- S.D.) of three independent experiments (done in duplicates) with significance (one-way ANOVA; *p≤0.05, **p≤0.01, ***p≤0.005, ns - not significant) shown relative to non-infected (NI) control and between indicated pairs. Infections involved EPEC and isogenic strains lacking a functional effector-delivery system (T3SS), Tir (*tir*) and/or its ligand Intimin (*eae*) and/or the NleB1/NleE1 (*nleB1*/*nleE1*) effectors.

Previous work [10] suggested that the Tir and NleH effectors are not required for EPEC to inhibit NF-κB activity, with recent studies reporting a non-essential role not only for NleH but also NleC [22κ27]. Indeed, HeLa cells infections with a *tir* mutant confirmed Tir's non-essential role in inhibiting TNFα-induced IL8 secretion [10] but also revealed a small, but statistically significant defect (Fig. 6A). To examine the relationship of this defect to the absence of Tir/Intimin-mediated intimate EPEC-host cell interaction, assays were carried out with an *eae* (Intimin-deficient) mutant. Unexpectedly, these studies indicated that Intimin (indirectly or directly) induces NF-κB activity or suppresses effector-mediated inhibitory mechanism(s), as the *eae* mutant inhibited TNFα-induced IL8 secretion to a greater extent than wild type EPEC (Fig. 6A). Intimin alters host cellular processes by Tir-dependent and -independent mechanisms [40]. Infection studies with an *eaetir* double mutant suggest that this Intimin function relates to Tir-dependent and -independent mechanisms (Fig. 6A).

Interestingly, time course infection studies support a Tir-TRAF2 relationship as EPEC induced a dramatic loss in the levels of activation-associated multimeric TRAF2 proteins by a process dependent on Tir and Intimin (Fig. 6B). The Intimin-dependent nature of this event, in contrast to that mediated by ectopically-expressed Tir (Fig. 2), implies that EPEC has evolved Intimin-dependent mechanisms for regulating this Tir activity. Interestingly, other effectors appear to contribute at early time points, with the *eae* mutant appearing to display an augmented ability to reduce TRAF2 levels (Fig. 6B). Intriguingly, confocal microscopy studies of disease-relevant polarised cells infected with EPEC only detect Tir at the apical (surface) membrane whereas a transient pool is evident within the cytoplasm of *eae* mutant-infected cells (Fig. S2). This suggests that Intimin promotes Tir's rapid association with the plasma membrane, with the transient cytoplasmic pool perhaps promoting Tir-TRAF2 interactions to explain the *eae* mutant's Tir-dependent augmented ability to reduce the level of TRAF2 multimers and inhibit TNFα-induced IL8 secretion.

Recent studies have described a prominent role for NleE1, promoted by NleB1 or NleC, in the EPEC NF-κB inhibitory process, as *nleCnleE1* and *nleB1nleE1* double mutants behaved like a T3SS-defective strain, compared with partial defects for single mutants [22], [25], [28], [29]. However, we and others have described T3SS-independent inhibitory mechanisms in the employed HeLa cell models (Fig. 1) [28], [29] that may obscure the contribution of effectors (Fig. 1) [28], [29]. Thus, an *nleB1nleE1* double mutant was generated and evaluated in a small intestinal model where EPEC was confirmed to inhibit NF-κB function solely in a T3SS-dependent manner [10] (Fig. 6C). Indeed, the *nleB1nleE1* double mutant behaved akin to the effector-delivery defective (T3SS) strain (Fig. 6C) despite displaying no obvious defect in delivering EspB or Tir effectors (not shown). By contrast, a *nleB1nleE1tir* triple mutant displayed a small (significant) capacity to inhibit NF-κB function - presumably due to remaining effectors. Interestingly, while EPEC and T3SS-mutant infected cells released ∼200 and ∼800 ng/ml of IL8, respectively, in response to TNFα treatment only ∼400ng/ml was secreted from *tir* mutant infected cells (Fig. 6C). Whilst these IL8 values support a non-essential contributory role for Tir in the inhibitory process, the difference between EPEC- and *tir*-infected cell was below the significance threshold (p = 0.075). Nevertheless, this work supports the idea that the NleB1/NleE1 effectors play a central role in enabling EPEC to inhibit NF-κB activity in intestinal cells, with the non-essential novel NF-κB inhibitory activities of NleC, NleH and Tir, presumably, playing evolutionary-advantageous roles in EPEC's lifecycle.

### Insights on NleB and NleE targets in NF-κB signalling

**Figure 7:**
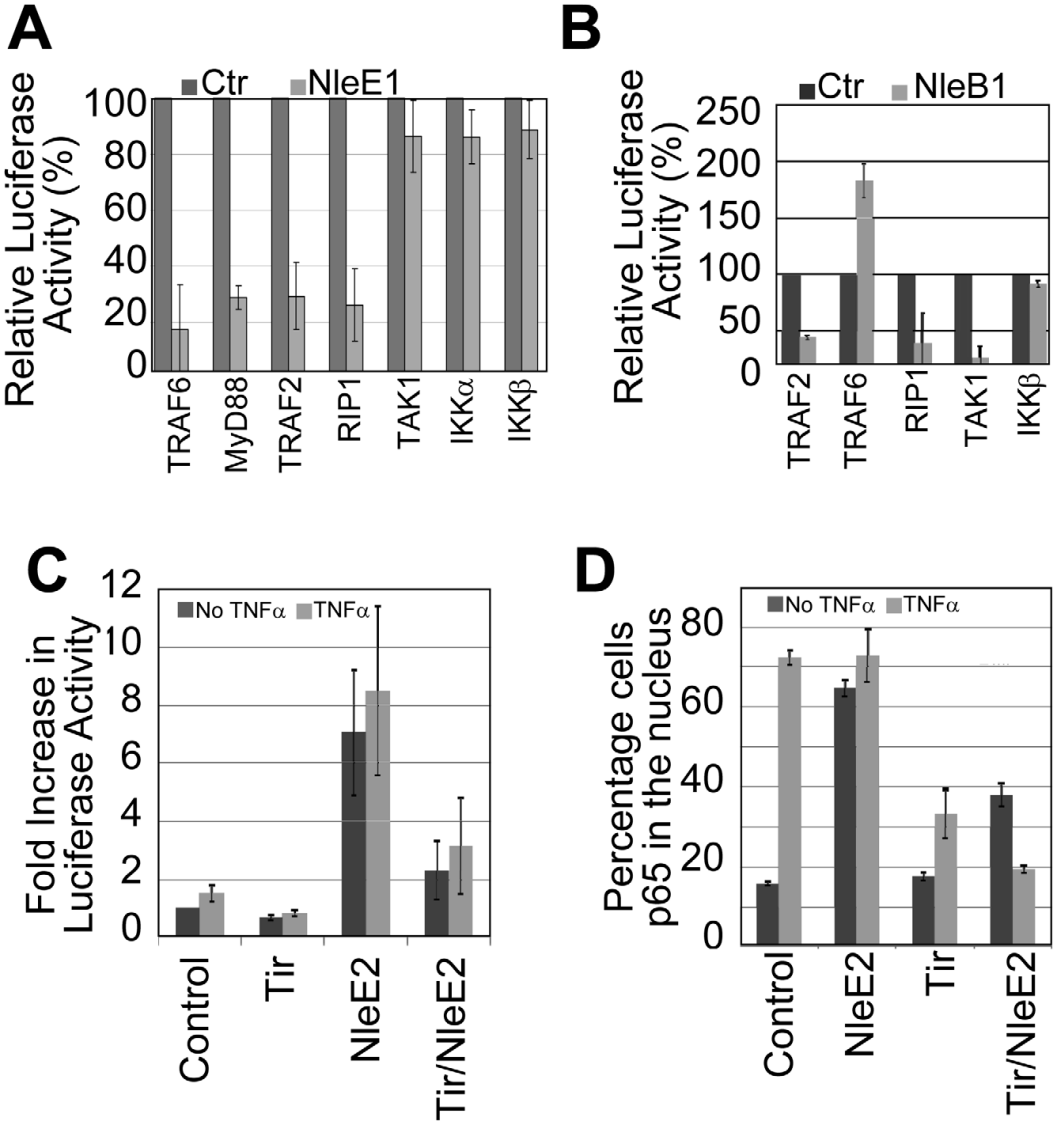
NleB1 and NleE1 targets in the subversion of NF-κB activity. HeLa cells were co-transfected with vectors encoding i) luciferase under the transcriptional control of NF-κB, ii) indicated signalling pathways components and iii) nothing (Ctrl empty plasmid), NleE1 (in A) or eGFP-NleB1 (in B) prior to assaying cellular luciferase activity. Relative luciferase activity, as percentage of control, is given as mean (+/- S.D.) of three independent experiments (done in duplicate; A) and one experiment (done in duplicate; B). In C and D, HeLa cells were transfected with Control (empty plasmid), NleE2 and/or Tir-eGFP encoding vectors prior to treating (light grey box) or not (dark grey box) with TNFα and processing for (C) luciferase activity - these experiments involved cotransfection with the NF-κB reporter vector or (D) microscopy-based quantification of the percentage of cells with p65 in the nucleus (counting a minimum of 50 cells). Data shown is mean (+/- S.D.) of three independent experiments with luciferase assays done in duplicate.

Given NleE1 and NleB1's key roles in the EPEC NF-κB inhibitory process, with only speculations on their targets [28], [29], we investigated where the blockage occurred by co-expressing them with signalling pathway components for NF-κB luciferase reporter assays. Expression of NleE1 inhibited NF-κB reporter activity driven by plasmid-expression of components from the TNFR (TRAF2, RIP1) and IL1R/TLR (TRAF6, MyD88) pathways (Fig. 7A) consistent with reports of it inhibiting NF-κB activation by multiple pathways [28], [29]. However, NleE1 failed to inhibit luciferase activity driven by plasmid-expression of TAK1 or IKK kinases (Fig. 7A) suggesting it inhibits TAK1 function to block signalling by TNFR, IL1R and TLR pathways. NleE1 may target TAK1 or factors needed for its activation, such as the TAB2/3 proteins which recruit TAK1 to ubiquitin-modified RIP1 and ubiquitin-modified TRAF6 proteins for activation in the TNFR and TLR/IL1R pathways, respectively [39]. By contrast, NleB1 inhibited luciferase activity driven by plasmid-expression of TRAF2 but not TRAF6 or IKKβ (Fig. 7B) supporting reports of it inhibiting signalling in TNFR but not TLR/IL1R pathways [28], [29]. Interestingly, NleB increased luciferase activity driven by TRAF6 suggesting that it has functions that (directly or indirectly) activate TRAF6-mediated signalling. Increases in TRAF6 signalling, despite NleB inhibition of TAK1 function (Fig. 7B), suggest that the effector may block RIP1-mediated activation of TAK1 to inhibit signalling in TNFR, but not TLR/IL1R pathways [39].

Interestingly, our screening program revealed that the NleE1 homologue, NleE2, induced NF-κB luciferase activity as effectively as TNFα(Fig. 7C) leading to a similar high level of p65 relocation into the nucleus (Fig. 7D). Indeed, both these NleE2-dependent alterations were inhibited by co-expressing Tir (Fig. 7C and 7D) suggesting that NleE2 activates NF-κB (either directly or indirectly) through a component at or upstream of Tir's target i.e. TRAF. While NleE2 is apparently not transferred into host cells [28], our finding supports the idea that EPEC effectors have features or functions that can activate NF-κB signalling and can be blocked by Tir.

## Discussion

In this study we describe a new property for the most-extensively studied EPEC effector by demonstrating that the Translocated Intimin receptor (Tir) protein has a potent and specific ability to prevent HeLa cells from activating NF-κB in response to the cytokine TNFα. Whilst this discovery involved ectopic expression of Tir and an indirect NF-κB reporter assay, its relationship to transcription factor function was demonstrated by several lines of evidence. Firstly, expression of Tir-eGFP, unlike eGFP, blocked the phosphorylation-associated activation of IKKα/β and the NF-κB component, p65 - events required for the nuclear import and transcriptional activity of NF-κB, respectively [13]. Importantly, absence of these modifications was not due to cell loss as Tir-eGFP expression had no observable impact on the total cellular level of IKK kinases or p65. Secondly, epifluorescent microscopy studies revealed that TNFα treatment induced some relocation of p65 into the nucleus of Tir-eGFP expressing cells but to a dramatically less degree than eGFP expressing cells. Indeed, as expected, these Tir-mediated inhibitory events translated into a dramatic deficiency in the NF-κB dependent event [35] of TNFα-augmented increases in IL8 secretion levels. Thirdly, use of a more physiologically relevant mechanism of introducing Tir into host cells (via the T3SS of another pathogen, *Yersinia*) inhibited TNFα-induced IL8 secretion to a similar level as control EPEC-infected cells, whereas the Tir-negative *Yersinia* strain had no inhibitory capacity. Finally, epifluorescent, biochemical and co-precipitation studies unearthed an inhibitory mechanism relating to Tir interaction with and subsequent cellular loss of a key component from the TNFR signalling pathway. These data illustrate that the EPEC Tir effector has a specific and potent ability to inhibit TNFα-induced NF-κB activation.

The absence of additional EPEC factors in these ectopic and *Yersinia*-delivery Tir experiments illustrate the Intimin-independent nature of the NF-κB inhibitory process. Whilst over a decade of studies has re-enforced the idea that Tir's subversive activities require it to interact with the EPEC surface protein, Intimin (and, thus, Tir's need to insert into the plasma membrane to act as a receptor for Intimin) a recent study described an Intimin-independent function [41]. Our discovery of a second such activity raises the possibility that Tir possesses additional Intimin-independent functions and the need to consider their contribution to Tir's critical role in the virulence of attaching and effacing pathogens that include strains targeting humans (EPEC and enterohaemorrhagic *E. coli*; EHEC), ruminants (EHEC) and various small mammals.

Our work also revealed a novel mechanism for a pathogen effector to inhibit NF-κB activity as it demonstrated that Tir interacts, directly or indirectly, with TRAF2 proteins (with a preference for activation-associated multimers) inducing the proteasomal-independent loss of this adaptor protein from host cells. TRAF adaptor proteins play critical roles in signalling to NF-κB by multiple pathways including the TLR, TNFR and IL1R pathways inhibited by EPEC [10,13,19κ21]. Indeed, Tir inhibited NF-κB reporter activity driven by plasmid-expression of the TRAF2 (Fig. 4) and TRAF6 (not shown) proteins of the TNFR and TLR/IL1R pathways, respectively. Moreover, TRAF2 and TRAF5 possess functionally redundant roles in TNFR-mediated signalling to NF-κB [37] suggesting that Tir also inhibits TRAF5 activity. It is speculated that Tir can inhibit signalling through other TRAF-dependent pathways. Six of the seven TRAF members carry amino-terminal zinc-binding motifs involved in their function as E3 ubiquitin ligases for activating downstream kinases, while TRAF1 (lacks the ‘Really Interesting New Gene’ RING domain) has regulatory functions [37], [42]. Studies with a dominant-negative variant of TRAF2 [43] suggests that Tir is unable to induce its degradation (Fig. S3) implicating a need for the absent RING domain in the degradation process. There are several examples of pathogens targeting TRAF proteins, including the poxvirus MC159 protein preventing TRAF2 sequestration into a signalosome [44] and the *Yersinia* YopJ effector deubiquitinating TRAF2 to inhibit signalling to NF-κB [45]. The *Yersinia* strain used in our studies has no detectable YopJ activity (linked to a polar insertion mutation of the *ypkA* gene immediately upstream of *yopJ*; Prof Hans Wolf-Watz personal communication). As far as we are aware, the proteasomal-independent degradation of TRAF2 by Tir represents a novel pathogen-mediated mechanism for inhibiting NF-κB activity. Determining if Tir induces the cellular loss of all or a subset of TRAF members may provide insights on the breadth of cytokine- and antigen-signalling pathways it can inhibit and/or highlight conserved features involved in the TRAF interaction and/or degradation processes. Studies are underway to define the features and mechanism by which Tir induces the proteasomal-independent degradation of TRAF adaptors.

Consistent with previous findings [10], Tir was not required for EPEC to inhibit TNFα-induced NF-κB activation though infection studies revealed a small, but statistically significant inhibitory defect for a Tir-deficient strain. This defect was unlinked to Tir's role with Intimin in mediating intimate EPEC-host cell interactions, as an Intimin-deficient (*eae*) mutant inhibited NF-κB activity to a greater extent than EPEC. This Intimin-related activity was associated with Tir-dependent and -independent mechanisms thereby revealing a new property for this EPEC surface protein. Intriguingly, strains lacking Intimin or Tir displayed a dramatic deficiency in EPEC's ability to decrease cellular levels of activation-associated TRAF2 multimers suggesting that, in the context of an EPEC infection, Tir requires Intimin to reduce TRAF2 cellular levels. Interestingly, microscopy studies identified a transient pool of Tir within the cytoplasm of epithelia infected with the Intimin-deficient, but not wildtype EPEC strain. While Tir has been proposed to insert into the plasma membrane during the translocation process [46], it is clear that it can insert from the host cytoplasm [47], though an infection-associated cytoplasmic pool has, until now, only been supported by Western blot analyses [34], [48]–[50]. It appears that Tir delivery into the host cytoplasm is normally followed by its rapid (Intimin signalling-promoted) association with the plasma membrane. The extended presence of Tir within the cytoplasm of *eae*-mutant infected cells may promote Tir-mediated loss of activation-associated TRAF2 multimers, as supported by the time course studies (Fig. 6B), to perhaps explain the Tir-dependent increased capacity of the *eae* mutant to inhibit TNFα-induced IL8 secretion.

While Tir's non-essential role in the EPEC NF-κB inhibitory process, like that of NleC and NleH [22]–[27], could be due to functional redundancy with other effectors, this is not the case as illustrated by studies with an *nleB1nleE1* double mutant. Thus, despite displaying no defect in delivering Tir into host cells, the double mutant had no significant ability to inhibit TNFα-induced IL8 secretion in HeLa or small intestinal models. This finding supports the reported key role for the NleE1/NleB1 effectors in blocking NF-κB function [25], [28], [29] and implies that the described novel NF-κB inhibitory activities of Tir, NleH and NleC effectors [22]–[27] (this study) are minor or transient during EPEC infections. As *Yersinia*-delivered and ectopically-expressed Tir proteins are potent inhibitors of TNFα-induced NF-κB activity, unlike EPEC-delivered Tir, this implies that EPEC possesses factors that suppress Tir's inhibitory function. Interestingly, ectopically-expressed Tir decreases TRAF2 cellular levels in an Intimin-independent manner (Fig. 5) while decreases mediated by EPEC-delivered Tir depend on Intimin (Fig. 6B). Ectopically-expressed (and *Yersinia*-delivered) Tir differs from the EPEC-delivered Tir by i) being mainly cytoplasmic ii) only undergoing partial host kinase-mediated modification and iii) failing to interact with Intimin [34] (Fig. S3). Thus, EPEC suppression of Tir's ability to decrease TRAF2 cellular levels (and presumably its ability to inhibit NF-κB activity) is linked to undefined EPEC factors enabling Tir to insert into the plasma membrane to interact with Intimin. Further studies are required to define the putative EPEC factors and mechanisms involved in this regulatory process.

Our screening program revealed NF-κB inhibitory activities for NleC [23] and Tir (this study), a NF-κB activatory function of NleE2 (this study) and confirmed inhibitory activities [22]–[29] for NleH (not shown), NleE1 (this study) and NleB1 (this study) effectors. By contrast, no significant NF-κB modulatory activity was evident from screening other LEE or non-LEE effectors, though it is possible that the findings included false negatives due to effector expression problems or expressing effector-fusion proteins. Indeed, our work supports the premise [22]–[27] that NleC and NleH inhibit NF-κB function by targeting components downstream of the IKK complex [23] (not shown), with the inhibitory activities of NleE1, NleB1 and Tir linked, respectively, to blocking the function of the TAK1, RIP1 and TRAF components upstream of IKK. Interestingly, while NleB1 inhibited signalling by TNFR pathway components, it promoted that mediated by the TLR/ILIR pathway protein, TRAF6 (but not downstream RIP1) suggesting that it has properties that induce TRAF6-mediated NF-κB activation. Indeed, the idea that EPEC effector features or properties can activate NF-κB is supported by the finding that ectopically-expressed NleE2 was as effective as TNFα at inducing p65 nuclear relocation. Interestingly, this NleE2-mediated event was blocked by co-expression of Tir suggesting that its activatory property, as per NleB1, is transmitted through TRAF proteins - a defined target of Tir to inhibiting signalling to NF-κB.

Our findings on LEE and Nle effectors, in light of published work, lend themselves to speculations on the evolution of EPEC's capacity to inhibit NF-κB. Genome sequencing projects suggest that pathogenic *E. coli* evolved from commensal *E. coli* through the horizontal-acquisition of new functions encoded on mobile genetic elements. Thus, enterotoxigenic *E. coli* (ETEC) virulence is linked to strains acquiring functional enterotoxins and an enterocyte-binding pilus [51], whilst that of EPEC is linked to the acquisition of the effector-encoding mobile genetic elements [4], [52]. As Nle effector genes are generally missing from non-pathogenic *E. coli* strains and require the LEE T3SS for delivery into host cells, it is reasonable to assume that the progenitor EPEC strain possessed the LEE, but not Nle-encoding genetic elements. EPEC factors (including flagella) and LEE subversive functions (eg disrupting cell-cell interactions) can activate NF-κB to induce the expression of anti-microbial and inflammatory molecules that inhibit EPEC's virulence-critical ability to colonise epithelia [10], [21]. Thus, the LEE region presumably encoded factor(s) to inhibit this event, with our screening program defining Tir as the only LEE effector with significant NF-κB inhibitory activity. Indeed, our definition of Tir-TRAF interactions within the cytoplasm to inhibit NF-κB activity may explain why Tir transits through this host compartment prior to inserting into the host membrane. It is possible that Tir's inability to completely block signalling-induced relocation of NF-κB into the nucleus (Fig. 2C) provided a selective advantage to strains acquiring mobile genetic elements expressing effectors that promote the inhibitory process (eg NleC degradation of nuclear NF-κB complexes). Undoubtedly, the key point in the evolutionary process relates to the acquisition of the NleB1/NleE1-encoding mobile genetic element (Integrative element 6; IE6) given their critical roles in blocking NF-κB activation in HeLa cells [25], [28], [29] and disease-relevant small intestinal models. This premise is supported by the *nleB1nleE1* genes being among the subset of *nle* genes found in all sequenced LEE-encoding pathogens [52] and their presence at the 3′ end of the LEE region in some enterohemorrhagic *E. coli* strains [53]. It is possible that this LEE/IE6 hybrid represents a minimal genetic unit required to provide strains with EPEC-like enteric pathogenic properties.

## Materials and Methods

### Bacterial strains, growth condition and cell culture

These studies used nalidixic acid resistant EPEC strains, specifically wild-type EPEC (E2348/69), *eae* (Intimin-deficient) and *espA* (T3SS-deficient) isogenic strains [54], [55]. The *nleB1nleE1* double mutant was generated using described standard allelic exchange procedures [56], [57] to remove (confirmed by PCR analyses) the entire gene sequence (and inter-gene region) of the adjacent *nleB1* and *nleE1* genes. The *nleB1nleE1tir* triple mutant was generated using an available *tir*-deletion suicide vector as described [57]. Strains were grown in Luria-Bertani (LB) broth containing nalidixic acid (25 ug/ml final conc.) from single colonies, without shaking, at 37°C in a 5% CO_2_ incubator overnight. The *Yersinia* strains and their usage was as previously described [34]. Hela cells (ATCC CCL2) were grown at 37°C with 5% CO_2_ in Dulbecco's Minimal Eagles Medium (DMEM) supplemented with 10% heat-inactivated foetal calf serum and 2 mM L-glutamine. Caco2 parental or TC7 subclone cells were seeded at confluence onto Transwells (Corning) and polarised over 12–15 days as previously described [5], [6].

### Plasmids

Prof Luke O'Neill (Trinity College, Dublin) kindly provided plasmids relating to the NF-κB luciferase-reporter construct and expression of IKKα, IKKβ, TRAF2, TRAF6, and MyD88 [30] with those for RIP1 [31] and TAK1 [32] kindly provided by Prof's Jürg Tschopp (University of Lausanne, Switzerland) and Martin Dorf (Harvard, USA), respectively. Tir-eGFP, eGFP-NleB1, NleE1 and NleE2 proteins were expressed from p*EGFP*-N1 (Clontech), p*EGFP*-C1 (Clontech), pIRES (Clontech) and pcDNA3 (Invitrogen) vectors, respectively.

### Luciferase reporter assay

Hela cells (∼2×10^5^) seeded in 24-well plates were transfected the following day using JetPrime reagent (PEQLAB Ltd, UK) with a total amount of 250 ng DNA, comprising 100 ng of the NF-κB firefly luciferase reporter plasmid [30], 40 ng of the *Renilla reniformis* luciferase plasmid plus 110 ng of empty, or effector gene-containing plasmid. Levels of firefly luciferase expression were normalised against Renilla luciferase activity as a control for transfection efficiency (expressed as fold increase in luciferase activity over unstimulated control cells). When transfection efficiency was routinely found to be ∼65–80%, the *Renilla* luciferase plasmid was replaced with empty plasmid. High transfection efficiencies for p*EGFP* and/or p*EGFP-tir* experiments were routinely verified by visualising the eGFP signal. Twenty four hours post transfection, cells were incubated with or without TNFα (10 ng/ml) for 30 minutes, lysed in 100 µl of passive lysis buffer (Promega Ltd, Southampton, UK) for 15 minutes at room temperature with cell extracts taken for assessment of firefly luciferase activity following standard protocols and a FLUOstar Optima 413-3266 plate reader (BMG Labtech, Germany).

### Infection protocols

LB grown EPEC cultures were first diluted (1∶10) in DMEM and incubated for 3 hours at 37°C in a 5% CO_2_ incubator. The typical optical density (OD_600_) was between 0.2–0.3 with infections carried out at a multiplicity of infection, MOI, of ∼100∶1. The HeLa cell medium was replaced with DMEM at least 2 hours prior to infection (routinely 3 hours unless stated otherwise), with studies on transfected cells normally 24 hours post-transfection. *Yersinia* YIII MEKA strains were grown in modified brain-heart medium supplemented with 20 mM MgCl_2_ and 5 mM EGTA at 26°C without shaking, and used for infections as previously described [34]. When appropriate, cells were incubated with bactericidal levels of gentamycin (100 µg/ml final conc.) for 1 hour prior to adding TNFα (10 ng/ml) for between 30 minutes (the routine) and up to 2 hours. EPEC infections did not induce significant cell detachment under the employed experimental conditions.

### Immunoblotting

HeLa cells were washed with cold Phosphate Buffered Saline pH 7.4 (PBS) and lysed with 1% Triton X-100 in the presence of protease inhibitors (1/1000 dilution, Sigma cocktail), sodium fluoride, sodium orthovanadate and PMSF (1.2, 1.2 and 1 mM final concentration, respectively). When appropriate centrifugation (13000 x g 5 minutes) was used to separate insoluble (contains host nuclei and cytoskeleton as well as adherent bacteria) and soluble (contains host cytoplasmic and membrane proteins as well as T3SS-delivered proteins). Samples were resolved on 10% SDS PAGE, transferred onto nitrocellulose, blocked in 5% Blotto milk powder/PBS/0.02% Tween and probed with antibodies against IKKα/β (Santa Cruz), phospho IKKα/β (Cell Signaling), NF-κB p65 (Santa Cruz), phospho p65 (Ser536), TRAF2 (Cell Signaling), actin (Sigma), FLAG tag (Sigma), Myc tag (generous gift; Prof D. Mann, Newcastle University) or GFP (Zymed). Absence of reducing agents allowed the detection of TRAF2 multimeric bands. Primary antibodies were incubated overnight in a 5% bovine serum albumin (BSA)/PBS solution, washed extensively. Bound antibodies were detected using horseradish peroxidase-conjugated secondary antibodies and Super Signal West Pico chemiluminescent substrate (Pierce) with Hyperfilm ECL (Amersham Biosciences) following the manufacturer's recommendations.

### IL8 secretion assay

Supernatants (0.5 ml) were taken from above the HeLa cells and assayed for the level of IL8 using an ELISA kit (DB Biosciences) following the manufacturer's recommendations.

### Immunoprecipitation studies

Immunoprecipitation of Tir-eGFP was performed using GFP-Trap A beads (Chromotek) according to the manufacturer's instructions. Briefly, 24 hours post-transfection, Hela cells were lysed in RIPA buffer, centrifuged and the supernatant incubated with GFP-Trap beads for 30 minutes at 4°C. Following centrifugation, the unbound material was harvested and the beads washed before being resuspended in sample SDS buffer for Western blot analyses.

### Immuno-fluorescence microscopy

Following experimentation, Hela cells seeded on glass coverslips were washed three times with PBS prior to fixing (2.5% Paraformaldhyde - Sigma - in PBS) for 30 minutes and permeabilisation of host membranes in Triton-X100/BSA (0.1% and 2.5% final conc, respectively)/ PBS solution for 30 minutes. Cells were then incubated overnight in the fridge with an appropriate primary antibody in 2.5% BSA/PBS solution, followed by multiple PBS washes and incubation with Alexa 488 or Alexa 555-conjugated secondary antibodies in a 2.5% BSA/PBS solution (1 hour; room temperature). Washed cells were mounted in DAPI-Vectashield (Vector Laboratories) and examined on a Zeiss Axioskop Epifluorescent or a Leica TCS SP2UV confocal microscopy. Nuclear p65 and TRAF clusters were counted in a semi-blind fashion i.e. slides were assessed without considering the slide order or orientation with obtained data mapped to labelling. For co-localisation studies, cells were visualised using the confocal microscope using an x63 objective lens with serial optical slices taken along the z-axis of cells within a field of view (∼20 cells), with signals analysed by Leica software and plotted to illustrate the degree of overlap.

## Supporting Information

Figure S1 **Near-complete and partial colocalisation of Tir with IKKβ and TRAF2 proteins, respectively, with Tir expression linked to disruption of TRAF2 clusters.** HeLa cells were co-transfected with vectors encoding Tir, IKK**β** and/or TRAF2 (see Materials and Methods) prior to fixing (∼24 hour post-transfection) and examining the cellular location of Tir (GFP signal; Green), TRAF2 or IKK (appropriate primary antibody and fluorescent-conjugated secondary antibodies; Red) and nuclear DNA (via DAPI; blue). Viewing of signals (via a Leica SP2 confocal microscope) reveals in A) diffuse patterns for Tir (Green) and IKKβ (Red) with very high levels of co-localisation (yellow/orange colour in overlap), B) the presence of distinct cytoplasmic-located TRAF2 clusters (Red) with C) co-localisation of Tir with TRAF2 clusters (yellow/orange colour in overlap) linked to their disaggregation.
(TIF)

Figure S2 **Absence of Intimin leads to a detectable pool of Tir within the host cytoplasm of infected polarised cells.** Cells of the Caco-2 subclone, TC7, were seeded at confluence onto Transwells (Corning) for polarisation over 12–15 days. EPEC strains (pre-activated in DMEM at 37°C) were infected at an MOI of 1∶200 using gentle centrifugation (500 xg, 5 minutes) to initiate and synchronise EPEC-apical surface interactions. At indicated time points, the cells were washed and processed for microscopy as described [6]. Cells infected with wildtype EPEC (A) or the Intimin-deficient (*eae*) mutant (B) were stained to detect Tir (Green; anti-Tir antibodies), filamentous actin (Red; TRITC-phalloidin) and DNA (DAPI) prior to viewing on a Leica SP2 confocal microscope. Cells infected with a *tir*-deficient mutant were used as the negative control to ensure background signals were zero for the wild type EPEC and *eae* (Intimin-deficient) mutant infected cells. Images show the xz-axis of the monolayer with images representative of those obtained from two independent experiments.
(TIF)

Figure S3 **Tir does not induce the cellular loss of a dominant-negative variant of TRAF2.** Hela cells were transfected with vectors encoding TRAF2 or a dominant-negative variant [43] along with vectors encoding eGFP or eGFP-Tir prior to isolating total cellular extracts (24 hr post-transfection) and probing for TRAF2, Tir and actin (latter as a loading control). Arrow indicates a host-kinase modified Tir form, as reported [34].
(TIF)
