# Supplementary figures and images for: The Enteropathogenic E. coli (EPEC) Tir Effector Inhibits NF-κB Activity by Targeting TNFα Receptor-Associated Factors
Source: PLoS Pathog. 2011 Dec 1;7(12):e1002414. doi: 10.1371/journal.ppat.1002414 (PMC3228809; doi:10.1371/journal.ppat.1002414)

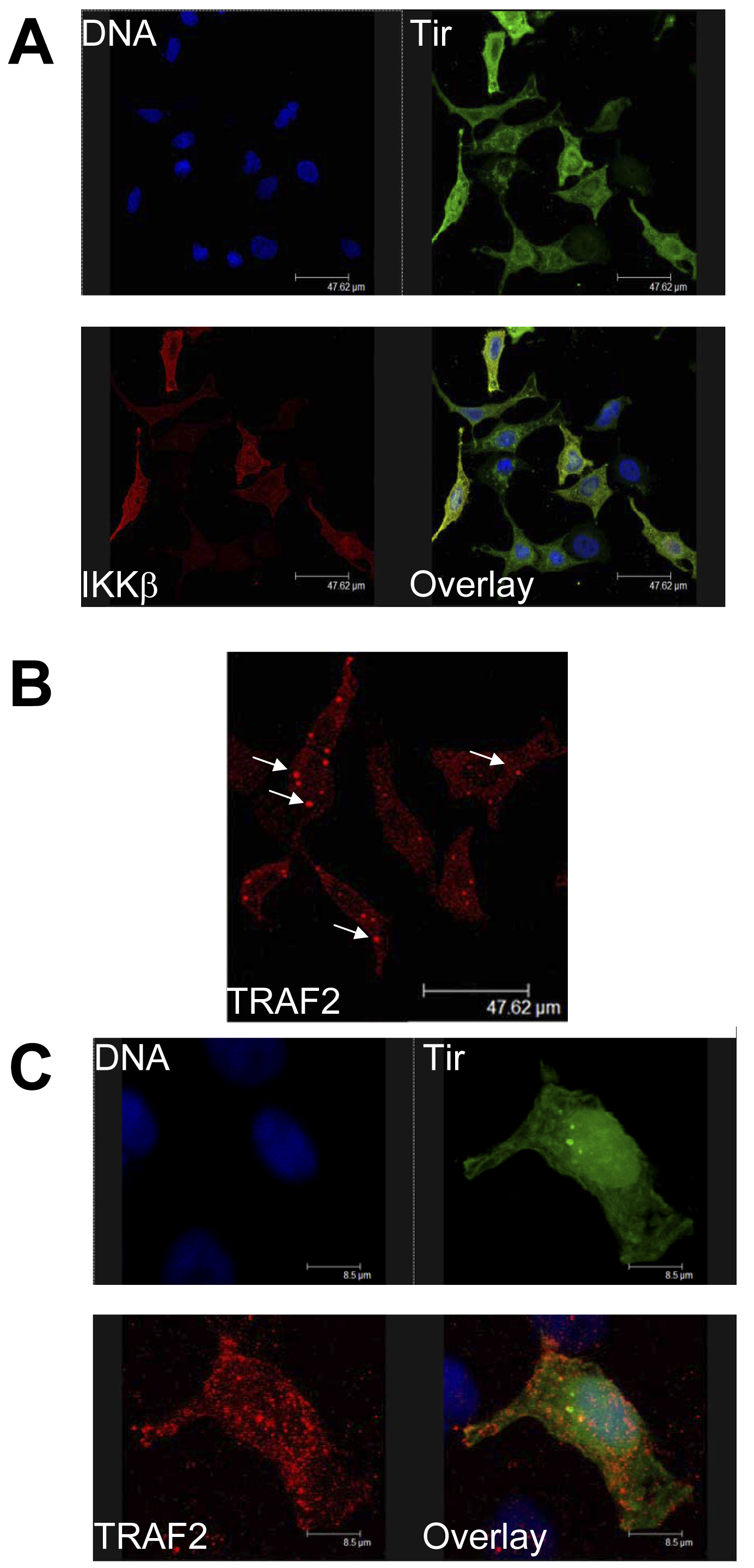

Supplement: Figure S1 — Near-complete and partial colocalisation of Tir with IKKβ and TRAF2 proteins, respectively, with Tir expression linked to disruption of TRAF2 clusters. HeLa cells were co-transfected with vectors encoding Tir, IKKβ and/or TRAF2 (see Materials and Methods) prior to fixing (∼24 hour post-transfection) and examining the cellular location of Tir (GFP signal; Green), TRAF2 or IKK (appropriate primary antibody and fluorescent-conjugated secondary antibodies; Red) and nuclear DNA (via DAPI; blue). Viewing of signals (via a Leica SP2 confocal microscope) reveals in A) diffuse patterns for Tir (Green) and IKKβ (Red) with very high levels of co-localisation (yellow/orange colour in overlap), B) the presence of distinct cytoplasmic-located TRAF2 clusters (Red) with C) co-localisation of Tir with TRAF2 clusters (yellow/orange colour in overlap) linked to their disaggregation. (TIF) [file ppat.1002414.s001.tif]

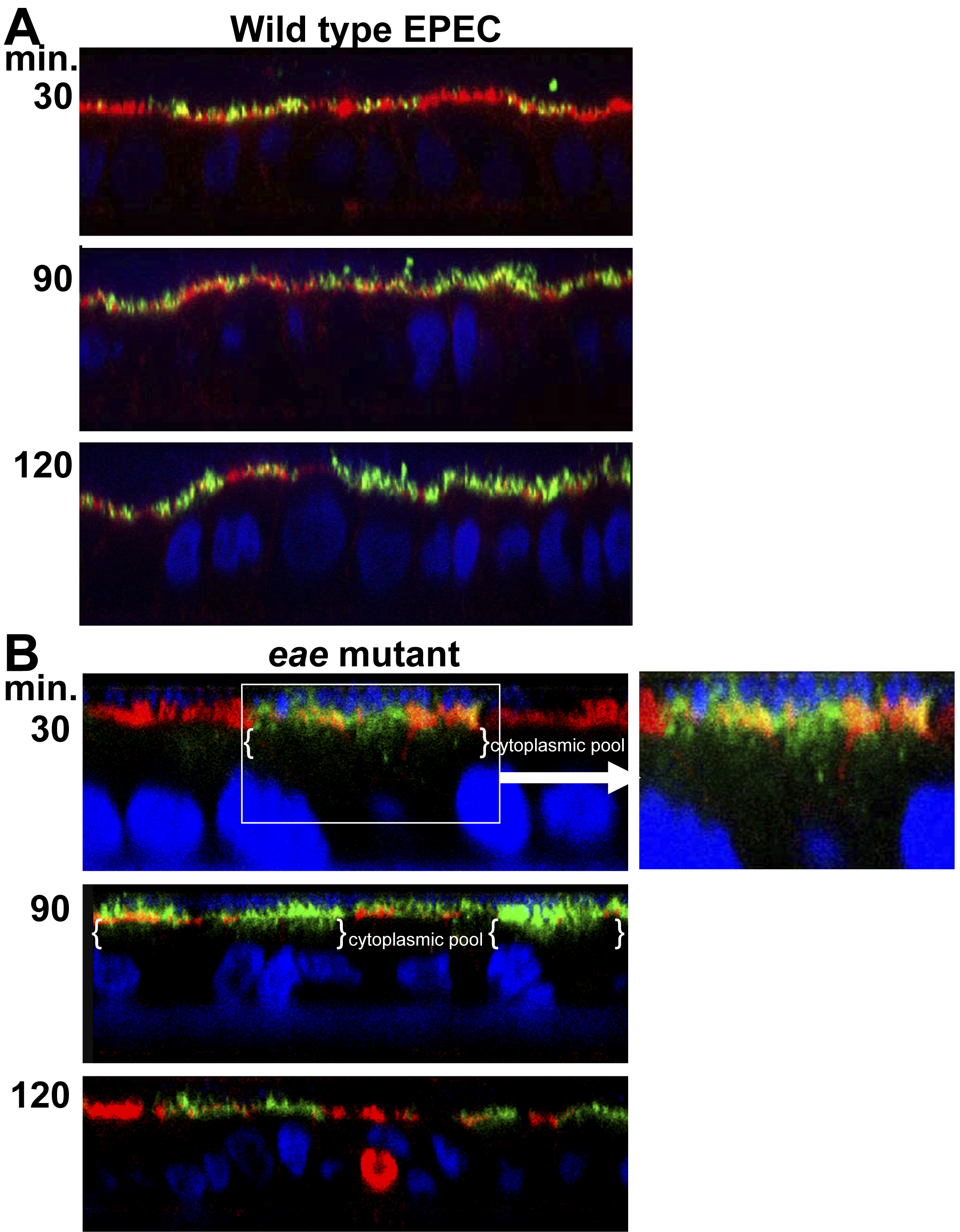

Supplement: Figure S2 — Absence of Intimin leads to a detectable pool of Tir within the host cytoplasm of infected polarised cells. Cells of the Caco-2 subclone, TC7, were seeded at confluence onto Transwells (Corning) for polarisation over 12–15 days. EPEC strains (pre-activated in DMEM at 37°C) were infected at an MOI of 1∶200 using gentle centrifugation (500 xg, 5 minutes) to initiate and synchronise EPEC-apical surface interactions. At indicated time points, the cells were washed and processed for microscopy as described [6]. Cells infected with wildtype EPEC (A) or the Intimin-deficient (eae) mutant (B) were stained to detect Tir (Green; anti-Tir antibodies), filamentous actin (Red; TRITC-phalloidin) and DNA (DAPI) prior to viewing on a Leica SP2 confocal microscope. Cells infected with a tir-deficient mutant were used as the negative control to ensure background signals were zero for the wild type EPEC and eae (Intimin-deficient) mutant infected cells. Images show the xz-axis of the monolayer with images representative of those obtained from two independent experiments. (TIF) [file ppat.1002414.s002.tif]

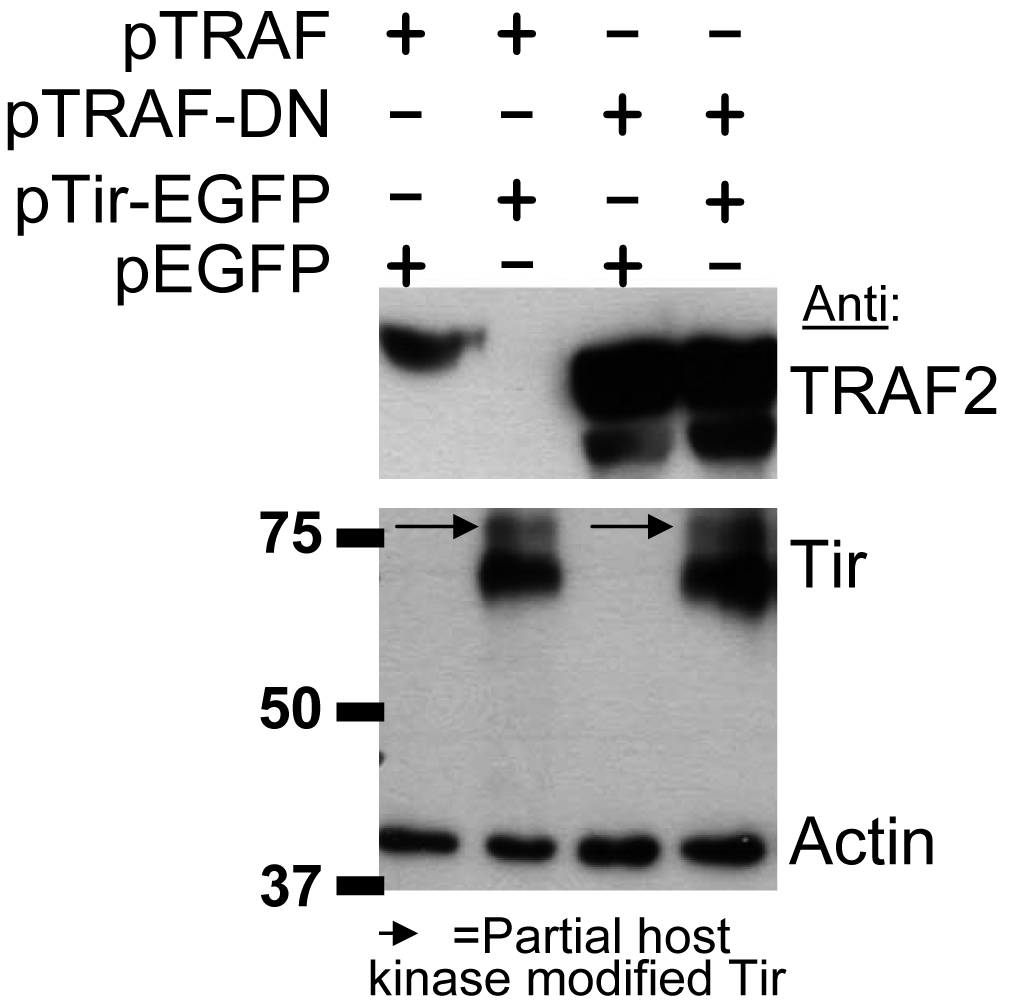

Supplement: Figure S3 — Tir does not induce the cellular loss of a dominant-negative variant of TRAF2. Hela cells were transfected with vectors encoding TRAF2 or a dominant-negative variant [43] along with vectors encoding eGFP or eGFP-Tir prior to isolating total cellular extracts (24 hr post-transfection) and probing for TRAF2, Tir and actin (latter as a loading control). Arrow indicates a host-kinase modified Tir form, as reported [34]. (TIF) [file ppat.1002414.s003.tif]
